# Synergistic Effects of Pre-Stretching and Aging Temperature on Precipitation Behavior and Damage Tolerance of an Al-Cu-Li Alloy

**DOI:** 10.3390/ma19061245

**Published:** 2026-03-21

**Authors:** Ben Lin, Changlin Li, Xiwu Li, Yongan Zhang, Kai Wen, Ying Li, Lizhen Yan, Yanan Li, Hongwei Yan, Zhihui Li, Baiqing Xiong

**Affiliations:** 1 State Key Laboratory of Nonferrous Structural Materials, China GRINM Group Co., Ltd., Beijing 100088, China; linben860620@163.com (B.L.); wenkai@grinm.com (K.W.); liying@grinm.com (Y.L.); yanlizhen@grinm.com (L.Y.); liyanan@grinm.com (Y.L.); yanhongwei@grinm.com (H.Y.); lzh@grinm.com (Z.L.); xiongbq@grinm.com (B.X.); 2 General Research Institute for Nonferrous Metals, Beijing 100088, China; 3 China Academy of Launch Vehicle Technology, Beijing 100076, China; 4 GRIMAT Engineering Institute Co., Ltd., Beijing 101407, China

**Keywords:** Al-Cu-Li alloy, pre-stretching deformation, aging temperature, damage tolerance

## Abstract

This study systematically investigates the synergistic effects of the pre-stretching deformation and aging temperature on the precipitation behavior and mechanical properties of an Al-Cu-Li alloy. The results indicate that increasing the pre-stretching deformation significantly refines and increases the number density of *T*_1_ and *θ*′ phases while optimizing the grain boundary precipitate morphology, thereby enhancing the fracture toughness and fatigue resistance without compromising the high strength. In contrast, elevating the aging temperature promotes the coarsening of the *T*_1_ phase, inhibits *θ*′ precipitation, and coarsens the grain boundary precipitates, leading to a deteriorated damage tolerance. By matching 3.5~4.5% pre-stretching with 145~155 °C aging, a synergistic optimization of ultra-high strength and damage tolerance can be achieved.

## 1. Introduction

Lightweight high-strength aluminum alloys are increasingly utilized in aerospace, transportation, and other industries. Among them, Al-Cu-Li alloys are now considered ideal for aerospace structural components, offering a low density, high specific strength and stiffness, and favorable damage tolerance [1,2,3]. These modern Al-Li alloys, primarily strengthened by the *T*_1_ (Al_2_CuLi) phase, exhibit significant lightweighting advantages in load-bearing components such as the propellant tank and wing panels [4,5,6]. However, achieving ultra-high strength while maintaining fracture toughness and fatigue resistance remains a critical scientific challenge for the application of these alloys [7,8].

In Al-Cu-Li alloys, the primary strengthening phases include the *T*_1_ phase, the *θ*′ (Al_2_Cu) phase, and the S′ (Al_2_CuMg) phase [9]. The *T*_1_ phase exhibits a thin plate-like morphology, with a habit plane of {111}_Al_ and a semi-coherent interface with the Al matrix, enabling it to effectively impede the dislocation motion [10,11]. Cu and Li enhance the strength of the alloy by forming strengthening phases such as *T*_1_ (Al_2_CuLi) and δ′ (Al_3_Li). Mg, Ag, and Zn act synergistically to promote the nucleation and dispersed precipitation of the primary strengthening phase *T*_1_ (Al_2_CuLi), thereby significantly increasing the aging response rate and strength of the alloy. Mn and Zr, meanwhile, primarily contribute to grain refinement, the inhibition of recrystallization, and an improvement in the anisotropy and toughness through the formation of dispersoids such as Mn-containing phases or Al_3_Zr [12].

Research has demonstrated that the *T*_1_ phase strongly depends on crystal defects, particularly dislocations and subgrain boundaries. Consequently, introducing pre-stretching deformation has become an important processing method for regulating *T*_1_-phase precipitation. The pre-stretching process introduces abundant dislocations into the matrix, creating heterogeneous nucleation points that facilitate the precipitation of a finely dispersed *T*_1_ phase. Consequently, the alloy’s mechanical performance is significantly enhanced. However, the synergistic regulation mechanism of pre-stretching on both the *T*_1_ and *θ*′ phases, as well as its influence on the strength-toughness matching of the alloy, still requires in-depth investigation [13,14,15,16].

The aging temperature is another critical process parameter affecting the precipitation behavior. The aging temperature governs the atomic diffusion rate, nucleation driving force, and thermal stability of precipitates, consequently influencing their size, number density, and distribution characteristics [17,18]. Lower temperatures favor the formation of fine, dispersed precipitates, while higher temperatures promote precipitate growth and coarsening, potentially altering the grain boundary precipitation behavior and interfacial coherency, thereby significantly affecting the fracture toughness and fatigue properties [19,20,21]. Therefore, under the combined regulation of the pre-deformation and aging temperature, the competitive relationships between precipitates (*T*_1_ and *θ*′), the coordination mechanism between intragranular and intergranular precipitation, and their synergistic influence on damage tolerance require systematic investigation.

The 2055 alloy is developed with the objective of replacing the high-performance 7055 alloy, aiming to achieve a structural weight reduction while maintaining comparable or superior comprehensive properties. According to AMS 4257, its mid-composition limits are Al-3.7Cu-1.15Li-0.5Zn-0.45Ag-0.4Mg-0.3Mn-0.1Zr (in wt.%), and the extruded profiles exhibit a tensile strength of up to 600 MPa. However, research on the thick plates of this alloy remains limited, particularly regarding its damage tolerance performance, which warrants further investigation. In recent years, the increasing demands for damage tolerance in aerospace structures have drawn growing attention to Al-Li alloys, particularly regarding their fracture toughness and fatigue crack propagation behavior [22,23,24]. The damage tolerance performance depends not only on the strength level of the alloy but is also closely related to the microstructural characteristics. Studies have shown that fine, dispersed intragranular precipitates facilitate uniform deformation and delay void nucleation, while discontinuous grain boundary precipitates can effectively inhibit intergranular fracture and enhance the fracture toughness [25,26,27,28]. However, current research on the synergistic regulation of precipitation behavior in Al-Li alloys by pre-stretching and the aging temperature, and its impact on damage tolerance, remains insufficient, and the underlying mechanisms require further elucidation.

Based on the above considerations, we investigate an Al-Cu-Li alloy to systematically evaluate the effects of the pre-stretching levels (2.5%, 3.5%, and 4.5%) and aging temperatures (145 °C, 155 °C, and 165 °C) on the microstructural evolution, tensile properties, fracture toughness, and fatigue crack growth behavior. A detailed transmission electron microscopy (TEM) characterization was conducted to quantify the precipitation features, complemented by a fractographic analysis to elucidate the fracture mechanisms. The objective is to clarify the regulatory mechanisms of pre-stretching and the aging temperature on the competitive precipitation of the *T*_1_ and *θ*′ phases, grain boundary characteristics, and damage tolerance, thereby providing theoretical guidance for the heat-treatment optimization and engineering application of the investigated alloy.

## 2. Experimental

### 2.1. Material Preparations

The alloy was prepared by conventional melting methods. The raw materials consisted of commercial purity Al (99.9%), Li, Mg, Ag, and Zn (99.99%), as well as Al-Zr (5%) and Al-Mn (10%) master alloys. The alloy was melted using a non-vacuum resistance furnace and subsequently cast into a water-cooled copper mold to produce an ingot (200 mm × 200 mm × 50 mm). The actual chemical composition of the alloy is presented in Table 1. The ingot was subjected to homogenization heat treatment (440 °C/24 h + 505 °C/24 h), followed by scalping to remove the surface oxide layer. Subsequently, hot rolling was performed using a two-high reversing rolling mill. The rolling temperature was 420 °C, with a total deformation of 80%. The deformation per pass was 10%, and the sample was reheated in the furnace for 15 min between passes. The final thickness of the rolled plate was 10 mm. Homogenization and solution heat treatments were carried out in an air circulation box-type resistance furnace with a temperature deviation of less than 2 °C. The quenching medium was water at a temperature ranging from 20 to 25 °C, and the quench transfer time did not exceed 5 s. Aging treatments were conducted in a thermostatic oven, with a precision of ±1 °C.

### 2.2. Property Tests and Microstructure Observations

All experiments were conducted at room temperature, and three parallel specimens were tested for each condition. Tensile tests were conducted on an MTS CMC 4304 (Eden Prairie, MN, USA) universal testing machine at a constant strain rate of 2 mm/min, in accordance with GB/T 16865-2023 [28]. Round specimens with a parallel section diameter of 5 mm and a parallel section length of 25 mm were used. Fracture toughness testing was performed on an MTS-370.10 hydraulic servo fatigue-testing machine at room temperature with a loading frequency of 20 Hz, following the GB/T 42914-2023 standard [29]. Compact tension (CT) specimens with a thickness (B) of 10 mm, a width (W) of 20 mm, and an initial notch length (a_0_) of 9 mm were used, and the orientation was L-T. Fatigue crack growth rate tests were conducted on an MTS 370.10 hydraulic servo fatigue-testing machine according to the GB/T 6398-2017 standard [30]. Compact tension (CT) specimens with a thickness of 10 mm and a width of 50 mm were used, and the sampling orientation was L-T to evaluate crack propagation behavior along the specific orientation. The experiments were carried out with a loading frequency of 10 Hz and a stress ratio (R) of 0.1. Experimental data were processed using the seven-point incremental polynomial method. Microstructure observations for scanning electron microscope (SEM) were performed on a JEOL JSM 7900F field emission gun scanning electron microscope operating at 20 KV. The precipitates were characterized by transmission electron microscopy (TEM) in FEI Tecnai G2 F20 (Hillsboro, OR, USA) field emission transmission electron microscope operated at 200 kV. The samples for SEM characterization were mechanically ground and polished. TEM foils were prepared by mechanical grinding to a thickness of roughly 50 μm and subsequent twin-jet electropolishing. The process was carried out at –30 °C using a mixture of 30% nitric acid and 70% methanol.

## 3. Results

### 3.1. Mechanical Properties

**Figure 1:**
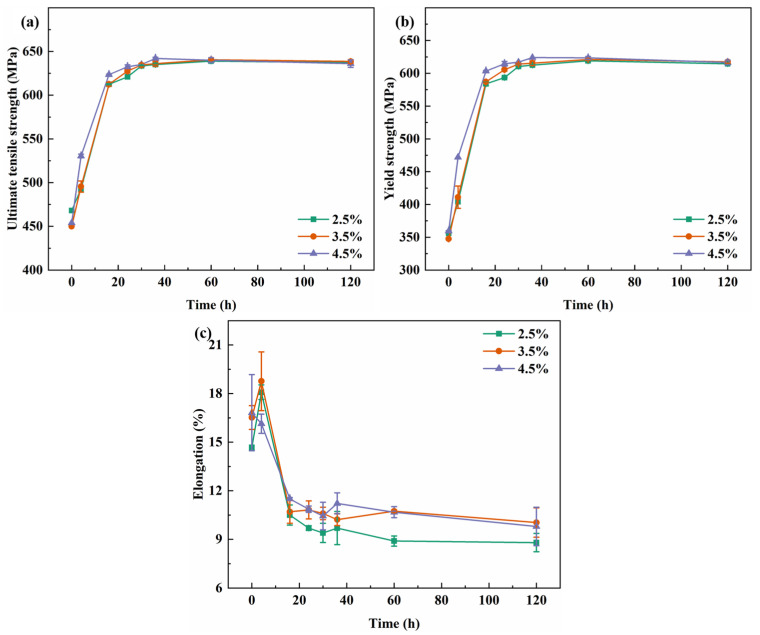
Tensile properties curves of alloy for different time after different pre-stretching deformation: (**a**) UTS; (**b**) YS; and (**c**) elongation.

Figure 1 illustrates the evolution of room-temperature tensile properties with the aging time at 155 °C for different pre-stretch levels (2.5%, 3.5%, and 4.5%). The tensile and yield strengths follow similar trends across all pre-stretch conditions: they increase rapidly in the early stages of aging, slow down after approximately 16 h, and peak between 30 h and 36 h. Beyond this period, further aging leads to a gradual decrease in strength. In contrast, elongation shows the opposite behavior, declining sharply initially and then decreasing more slowly before stabilizing with prolonged aging. A slight increase in both the tensile and yield strength is observed with increasing pre-stretch deformation. Based on these results, 30 h was identified as the peak-aging condition for all three pre-stretch levels. At this aging time, comparative investigations were carried out on the fracture toughness, fatigue crack propagation behavior, and precipitation characteristics of the alloys.

**Figure 2:**
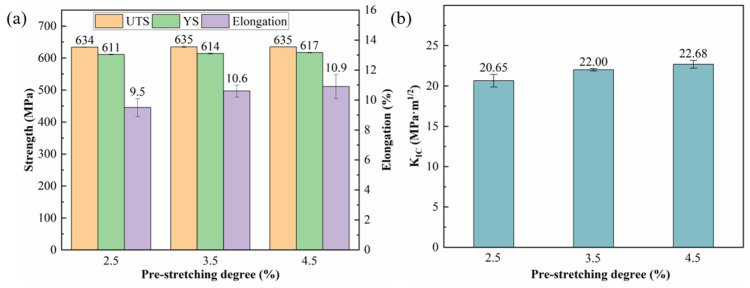
Mechanical properties values of alloy with different pre-stretching deformation: (**a**) tensile properties; and (**b**) fracture toughness.

Figure 2 shows the tensile properties and fracture toughness (K_IC_) of the alloy subjected to different pre-stretching deformations prior to aging at 155 °C for 30 h. It is found that the tensile strength remains largely unchanged across the range of pre-stretching levels applied, whereas the yield strength increases slightly. In contrast, the elongation exhibits a gradual rise with increasing pre-stretch deformation. Furthermore, the K_IC_ values also follow an upward trend, though the rate of improvement diminishes at pre-stretching levels of 3.5% and above.

**Figure 3:**
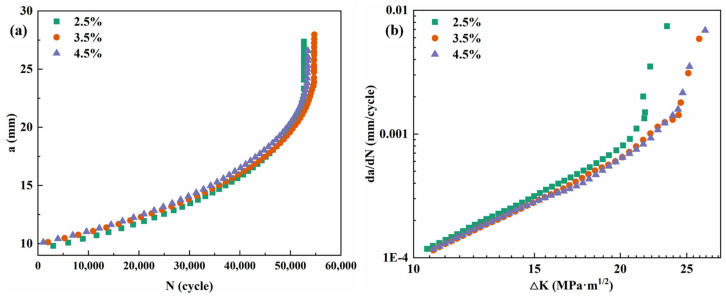
Fatigue crack growth curves of alloy under aging treatment at 155 °C/30 h after different pre-stretching deformation: (**a**) a-N curves; and (**b**) da/dN-ΔK curves.

Figure 3 presents the fatigue crack growth behavior of the alloy under different pre-stretching deformations followed by aging at 155 °C for 30 h. Minor differences are observed among the a-N curves across the various pre-stretching levels, whereas the da/dN-ΔK curves exhibit more distinct variations. As the deformation increases from 2.5% to 3.5%, the crack growth rate decreases notably in the medium-to-high ΔK regime. Further increasing the pre-stretch to 4.5% results in little additional change in the crack propagation rate. At ΔK ≈ 18 MPa·m^1/2^, the crack growth rate for the 2.5% pre-stretched alloy was 5.12 × 10^−4^ mm/cycle, compared with 4.95 × 10^−4^ mm/cycle for both the 3.5% and 4.5% conditions. This difference becomes more pronounced at ΔK ≈ 22 MPa·m^1/2^, where the growth rate reaches 1.70 × 10^−3^ mm/cycle for the 2.5% pre-stretched alloy, while remaining at only 1.1 × 10^−3^ mm/cycle for the 3.5% and 4.5% pre-stretched conditions.

**Figure 4:**
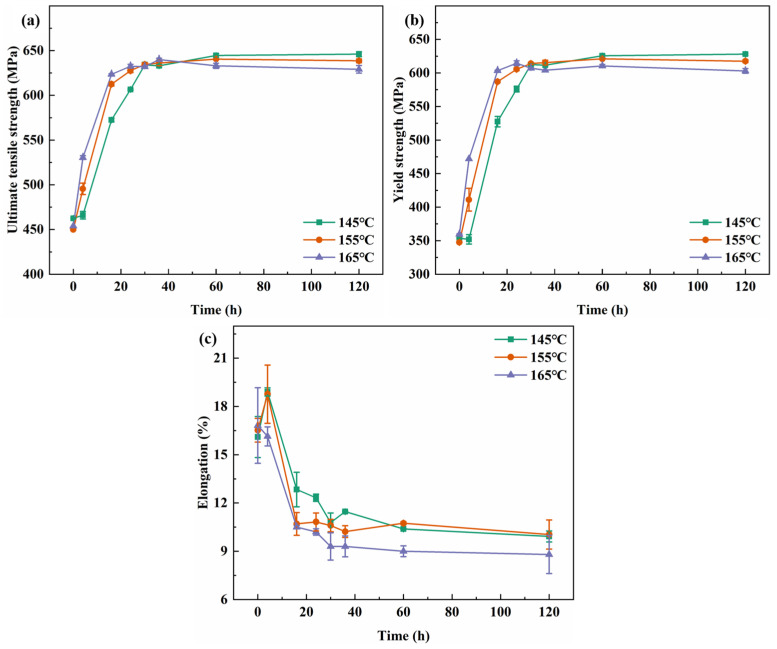
Tensile properties curves of alloy aged at different temperature for different time: (**a**) UTS; (**b**) YS; and (**c**) elongation.

Figure 4 presents the evolution of tensile properties over time for the alloy following a uniform 3.5% pre-stretching deformation and subsequent aging at various temperatures. As observed, the strength of the alloy displays similar trends with the aging time across different aging temperatures. They increase rapidly during the under-aging, slow down after approximately 16 h, and peak between 30 and 36 h. Moreover, a higher aging temperature leads to a faster attainment of the peak aging strength. Beyond this peak, a further extension of the aging time results in a gradual decline in strength. In contrast, the elongation exhibits an inverse relationship to the strength variations. It drops sharply in the early aging period, then continues to decrease more slowly before eventually stabilizing as the aging time proceeds.

**Figure 5:**
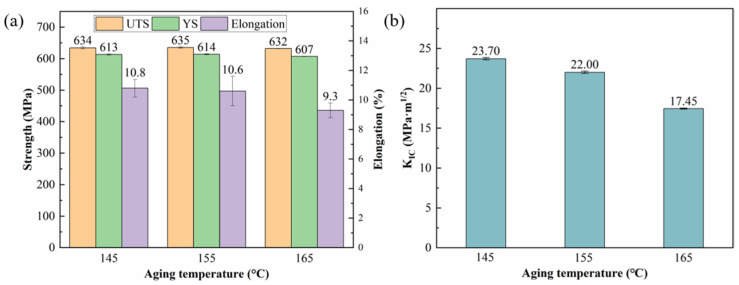
Mechanical properties values of alloy aged at different temperature for 30 h: (**a**) tensile properties; and (**b**) fracture toughness.

The tensile properties and fracture toughness values of the alloy after 30 h aging at different temperatures are shown in Figure 5. As the aging temperature increases, the strength of the alloy remains at essentially the same level. However, at 165 °C, both the yield strength and elongation exhibit a slight decline. In contrast, the fracture toughness decreases monotonically as the aging temperature rises. When the temperature increases from 145 °C to 155 °C, the K_IC_ value drops from 23.70·MPa·m^1/2^ to 22.00 MPa·m^1/2^. A further increase to 165 °C leads to a more pronounced reduction, with the K_IC_ value falling significantly to 17.45·MPa·m^1/2^.

**Figure 6:**
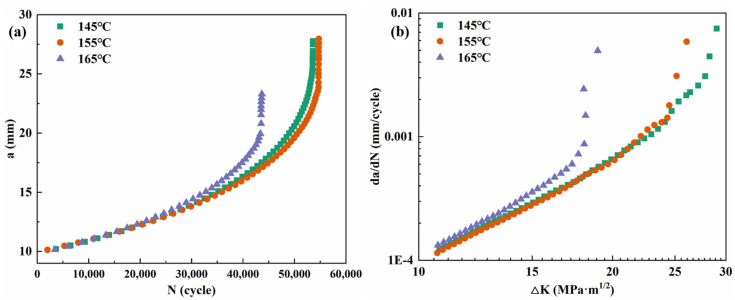
Fatigue crack growth curves of alloy aged at different temperature for 30 h: (**a**) a-N; and (**b**) da/dN-ΔK.

Figure 6 illustrates the fatigue crack growth curves of the alloy after the 3.5% pre-stretching deformation and 30 h of aging at different temperatures. It can be observed that the crack growth rates for samples aged at 145 °C and 155 °C show a minimal difference across the entire range of the stress intensity factor (ΔK), with the two curves nearly overlapping. However, when the aging temperature is raised to 165 °C, the crack growth rate increases rapidly within the medium ΔK range (15~18 MPa·m^1/2^), corresponding to the steady-state propagation stage. At ΔK ≈ 18 MPa·m^1/2^, the crack growth rates for the alloys aged at 145 °C and 155 °C were 4.73 × 10^−4^ mm/cycle and 4.95 × 10^−4^ mm/cycle, respectively, indicating little variation. In contrast, the alloy aged at 165 °C exhibited a significantly higher growth rate of 8.71 × 10^−4^ mm/cycle, which represents an 84.1% increase compared to the 145 °C condition.

### 3.2. Precipitation Behavior

**Figure 7:**
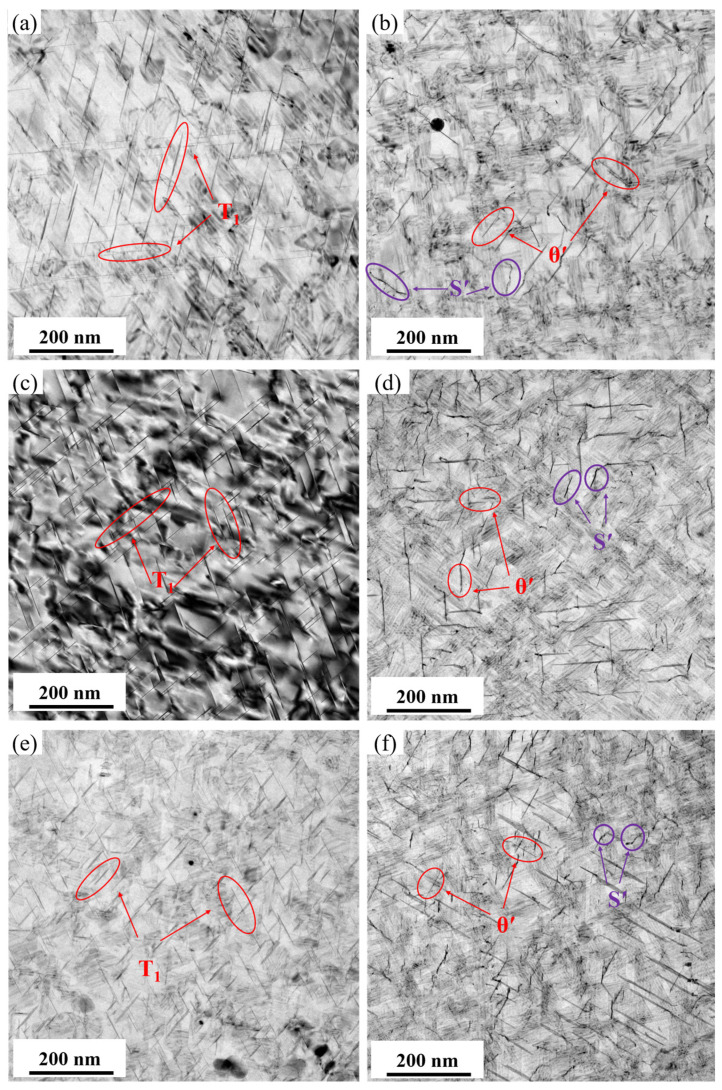
Bright-field TEM images of precipitates of alloy at 155 °C/30 h after different pre-stretching deformation: (**a**,**b**) 2.5%; (**c**,**d**) 3.5%; (**e**,**f**) 4.5%; (**a**,**c**,**e**) <110>_Al_ zone axis; and (**b**,**d**,**f**) <100>_Al_ zone axis.

**Figure 8:**
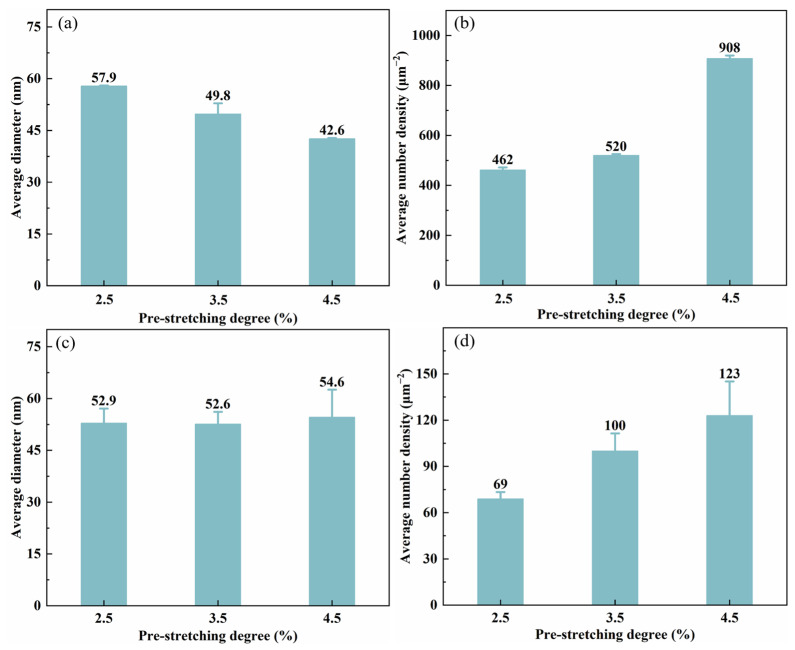
Statistic results of precipitation feature of *T*_1_ and *θ*′ precipitates under aging treatment at 155 °C/30 h after different pre-stretching deformation: (**a**,**b**) average diameter and number density of *T*_1_ phase; and (**c**,**d**) average diameter and number density of *θ*′ phase.

The TEM bright-field images in Figure 7 show the precipitates within the grains after aging at 155 °C for 30 h under different pre-stretching levels (2.5%, 3.5%, and 4.5%). The corresponding statistical results of the precipitates are presented in Figure 8. The alloy contains the primary strengthening phase *T*_1_, along with the secondary strengthening phases *θ*′ and S′. When observed along the <110>_Al_ zone axis, it is evident that, as the pre-stretching level increases, the size of the *T*_1_ phase progressively decreases while its number density continuously increases. A statistical analysis indicates that, at 2.5% pre-stretching, the *T*_1_ phase has an average diameter of 57.9 nm with a number density of 462 μm^−2^. When the pre-stretching increases to 3.5%, the average diameter decreases to 49.8 nm, while the number density rises slightly to 520 μm^−2^. With a further increase in pre-stretching to 4.5%, the average diameter declines linearly to 42.6 nm, accompanied by a significant increase in the number density to 908 μm^−2^. Similarly, an observation along the <100>_Al_ zone axis reveals that the influence of the pre-stretching level on the areal number density of the *θ*′ phase is similar to that on the *T*_1_ phase, whereas its effect on the precipitate size is not significant. According to the statistical results of the *θ*′ phase precipitation characteristics, as the pre-stretching level increases, the average diameter of the *θ*′ phase remains essentially constant at around 52 nm, while the areal number density increases monotonically and linearly from 69 μm^−2^ to 123 μm^−2^. These results demonstrate that increasing the pre-stretching level significantly affects the characteristics of the nanoscale precipitates in the alloy.

**Figure 9:**
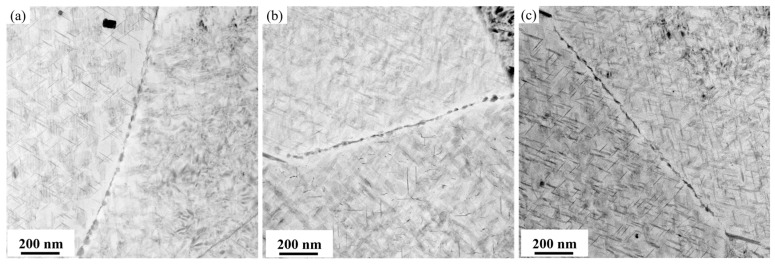
Bright-field TEM images of inter-granular precipitates of alloy under aging treatment.at 155 °C/30 h after different pre-stretching deformation: (**a**) 2.5%; (**b**) 3.5%; and (**c**) 4.5%.

Figure 9 presents bright-field TEM images showing large-angle grain boundary precipitates in the alloy after aging at 155 °C for 30 h under different levels of pre-stretching. It is observed that, as the pre-stretching level increases, the size of the grain boundary precipitates continuously decreases, and their distribution progressively shifts from continuous to discontinuous. Additionally, the width of the precipitate-free zone narrows. This trend is fully consistent with the characteristics of intergranular precipitation behavior. The greater pre-stretching deformation introduces a higher density of dislocations within the grains, which effectively promotes the intragranular precipitation of the *T*_1_ and *θ*′ phases. This process consumes a substantial number of solute atoms, thereby suppressing the coarsening of precipitates at the grain boundaries.

**Figure 10:**
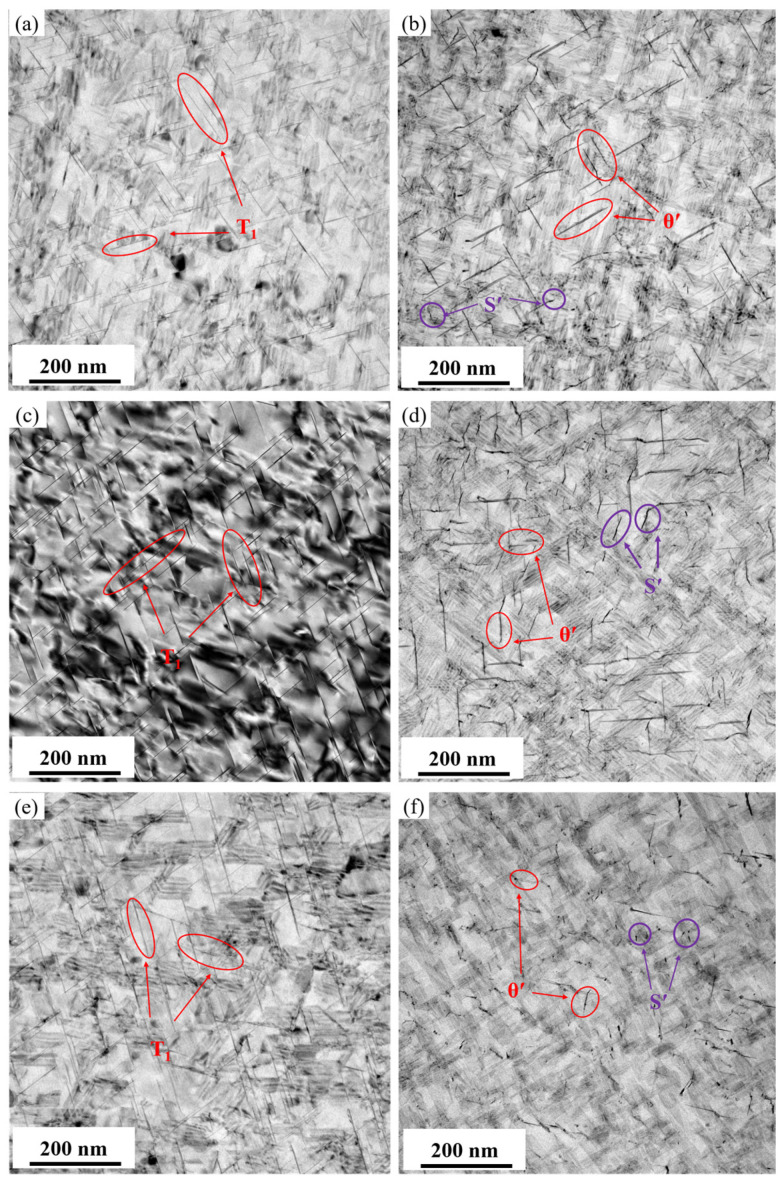
Bright-field TEM images of precipitates of alloys aged at different temperature for 30 h: (**a**,**b**) 145 °C; (**c**,**d**) 155 °C; (**e**,**f**) 165 °C; (**a**,**c**,**e**) <110>_Al_ zone axis; and (**b**,**d**,**f**) <100>_Al_ zone axis.

**Figure 11:**
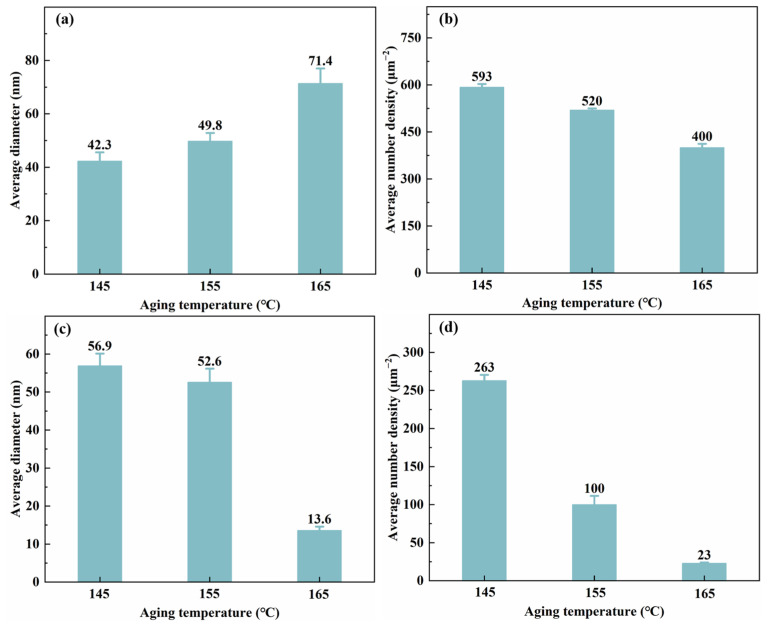
Statistic results of precipitation feature of *T*_1_ and *θ*′ precipitates of alloys aged at different temperature for 30 h: (**a**,**b**) average diameter and number density of *T*_1_ phase; and (**c**,**d**) average diameter and number density of *θ*′ phase.

Figure 10 displays the bright-field TEM images of intragranular precipitates after aging for 30 h at different temperatures (145, 155, and 165 °C) following a 3.5% pre-stretch deformation. Figure 11 provides the corresponding statistical results for the average diameter and number density of the precipitates. When observed along the <110>_Al_ zone axis, the *T*_1_ phase shows an increase in size but a decrease in quantity as the aging temperature rises. According to the statistical analysis, at 145 °C, the *T*_1_ phase has an average diameter of only 42.3 nm and an average area number density of 593 μm^−2^. When the aging temperature is increased to 165 °C, the average diameter grows to 71.4 nm, while the number density drops to 400 μm^−2^. The increase in average diameter was particularly notable when the aging temperature was raised from 155 °C to 165 °C. In contrast, when observed along the <100>_Al_ zone axis and combined with the statistical results of the *θ*′ phase precipitation characteristics, it is found that, as the aging temperature increases, the trend of the average number density of the *θ*′ phase changes similarly to that of the *T*_1_ phase, showing a monotonically decreasing trend from 263 to 23 μm^−2^. Meanwhile, the average diameter slightly decreases from 56.9 nm to 52.6 nm. At 165 °C, the average diameter significantly decreased to 13.6 nm. It is observed that, although the average diameters and number densities of the precipitated phases differ markedly at different aging temperatures, the differences in peak strength are relatively small. This indicates that the strength contributions of the different precipitated phases are essentially equivalent.

**Figure 12:**
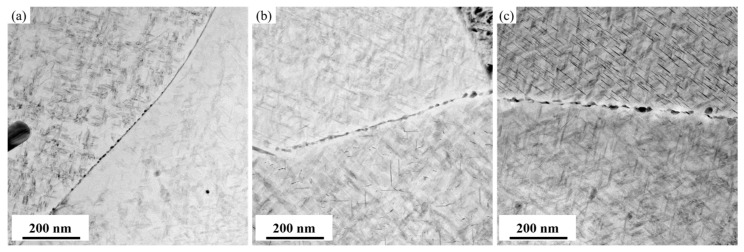
Bright-field TEM images of inter-granular precipitates of alloy aged at different temperature for 30 h: (**a**) 145 °C; (**b**) 155 °C; and (**c**) 165 °C.

Figure 12 shows bright-field TEM images of grain boundary precipitates in the alloy after aging at different temperatures for 30 h. It can be observed that, as the aging temperature increases, the size of the grain boundary precipitates continues to grow. Therefore, by combining the precipitation characteristics within the grains and at the grain boundaries, it can be concluded that increasing the aging temperature suppresses the nucleation of the *T*_1_ phase, while promoting its growth and coarsening. Simultaneously, it accelerates the nucleation and growth of grain boundary precipitates, consuming a large amount of Cu atoms. As a result, the precipitation behavior of the *θ*′ phase is significantly inhibited.

### 3.3. Fracture Morphology

**Figure 13:**
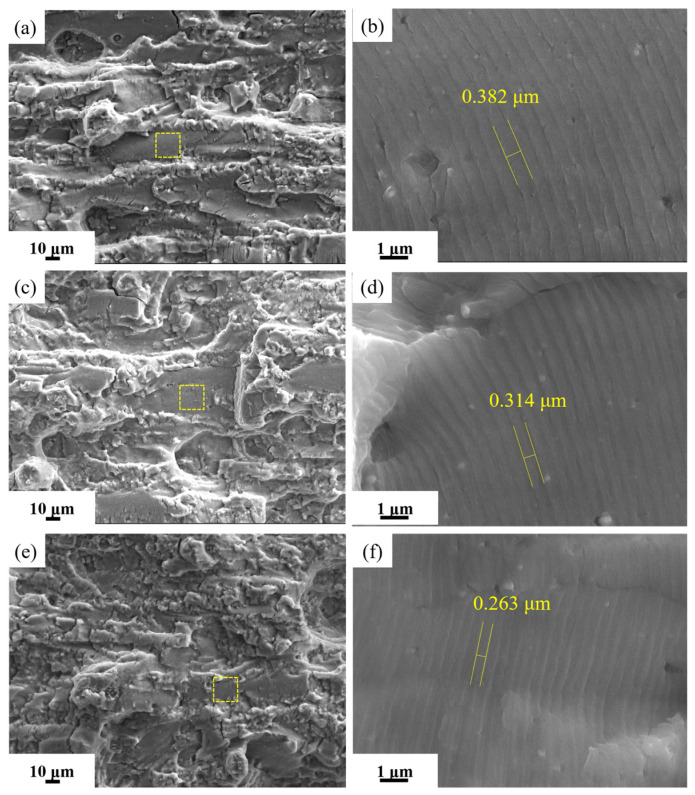
SEM images of fracture of fatigue crack growth samples of alloy under aging treatment at 155 °C/30 h after different pre-stretching deformation during steady expansion stage: (**a**,**b**) 2.5%; (**c**,**d**) 3.5%; and (**e**,**f**) 4.5% (ΔK ≈ 22 MPa·m^1/2^, a ≈ 11 mm).

**Figure 14:**
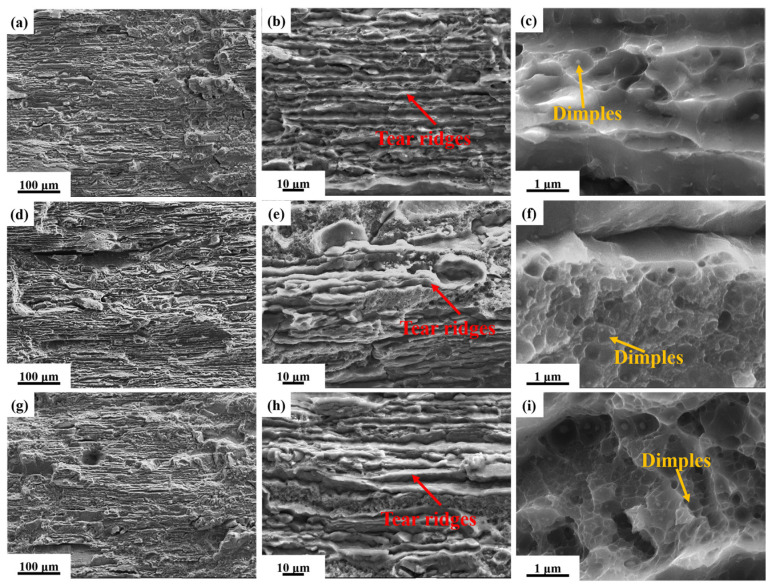
SEM images of fracture of fatigue crack growth samples of alloy under aging treatment at 155 °C/30 h after different pre-stretching deformation during unstable expansion stage: (**a**–**c**) 2.5%; (**d**–**f**) 3.5%; and (**g**–**i**) 4.5%.

Figure 13 shows the SEM images of fracture morphology at ΔK ≈ 22 MPa·m^1/2^. The low-magnification SEM image reveals that the fracture surface exhibits relatively distinct secondary cracks, tear ridges, and small planar facets. Under high magnification(yellow box), fatigue striations can be observed. At the same stress intensity factor range, the width of the fatigue striations gradually decreases with increasing pre-stretching deformation. Figure 14 shows the SEM images of fracture surfaces during the instability propagation stage. Low-magnification SEM reveals that, after 2.5% pre-stretching deformation, the fracture surface of the alloy tends to be flat with minimal surface roughness. As the pre-stretching deformation increases, the fracture surface becomes increasingly uneven with greater height variations. High-magnification SEM reveals that the proportion of ductile dimple zones increases with greater pre-stretching deformation, which is consistent with the enhanced fatigue crack propagation resistance of the alloy. Therefore, the optimal pre-stretching deformation range is 3.5~4.5%, achieving an optimal balance between strength and damage tolerance for the investigated alloy.

**Figure 15:**
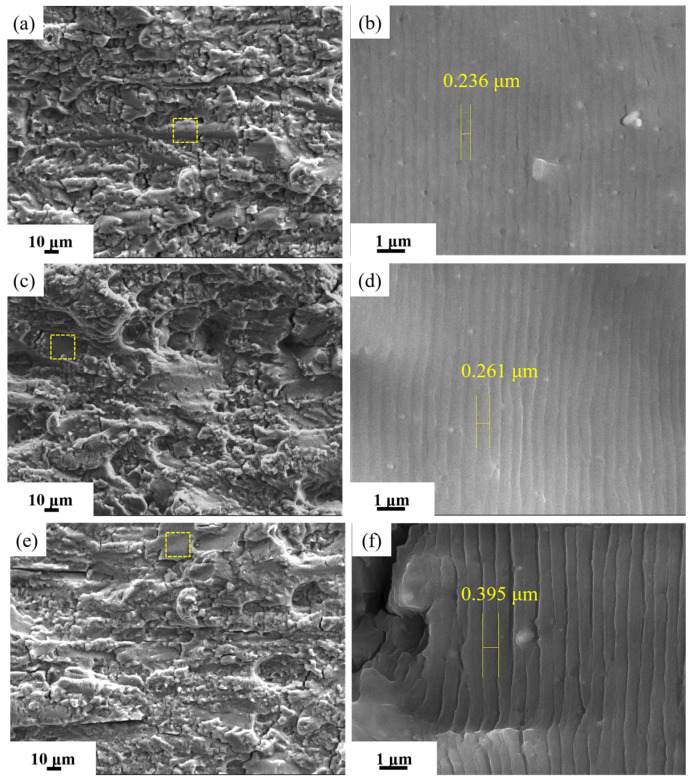
SEM images of fracture of fatigue crack growth samples of alloy aged at different temperature for 30 h during steady expansion stage: (**a**,**b**) 145 °C; (**c**,**d**) 155 °C; and (**e**,**f**) 165 °C (ΔK ≈ 18 MPa·m^1/2^, a ≈ 9 mm).

**Figure 16:**
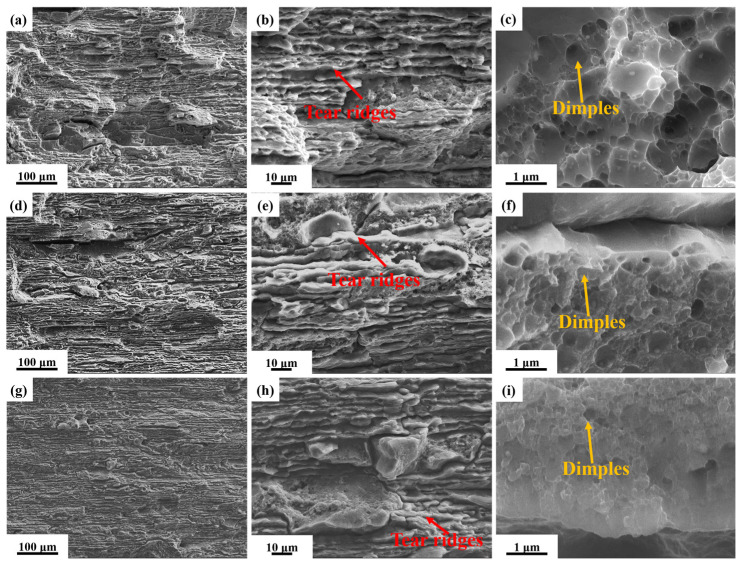
SEM images of fracture of fatigue crack growth samples of alloy aged at different temperature for 30 h during unstable expansion stage: (**a**–**c**) 145 °C; (**d**–**f**) 155 °C; and (**g**–**i**) 165 °C.

Figure 15 presents the corresponding fracture morphology at ΔK ≈ 18 MPa·m^1/2^. The low-magnification SEM observation reveals that the fracture surface contains relatively evident secondary cracks, tear ridges, and small planar facets. The corresponding high-magnification SEM image (yellow box) clearly shows striations formed during the crack propagation process. As the aging temperature increases, the striation width increases from 0.236 μm to 0.395 μm. Figure 16 presents SEM fractographs of the fracture surfaces from the unstable crack propagation stage in alloys subjected to different aging treatments. The low-magnification SEM images (Figure 16a,d,g) reveal that the fracture surface of the alloy treated at higher aging temperatures becomes relatively flat, with a noticeable reduction in surface height variation. Observations at medium magnification (Figure 16b,e,h) indicate that all alloys exhibit a mixed ductile–brittle fracture mode. However, the proportion of the dimple region continuously decreases with increasing aging temperature, while the proportion of cleavage steps formed by intergranular fracture increases. This suggests that a growing number of grain boundaries act as weak interfaces during the fracture process. A detailed examination of the dimple morphology, as shown in Figure 16c,f,i, demonstrates that, as the aging temperature rises, the dimples become smaller and more shallow, further corroborating the decline in toughness. Consequently, the optimal aging temperature range is identified as 145~155 °C, which ensures an ultra-high strength without significantly compromising the toughness, thereby achieving a better balance between strength and damage tolerance.

## 4. Discussion

### 4.1. Mechanism of Precipitation Modulation by Pre-Stretching

**Figure 17:**
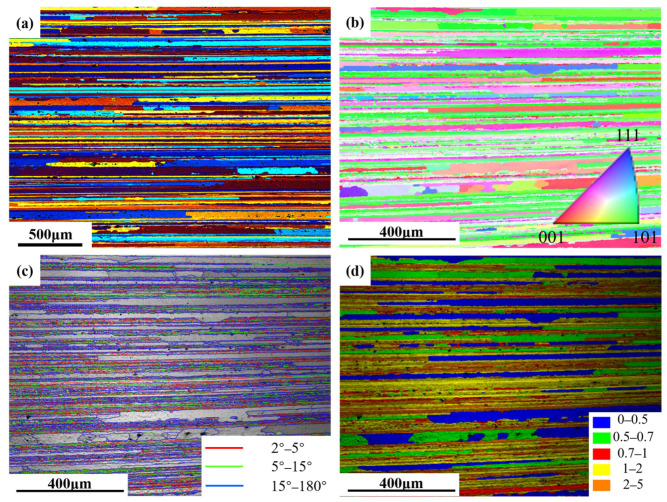
Polarized light OM and EBSD images of microstructure of the aged alloy: (**a**) polarized light micrography image; (**b**) auto grain image; (**c**) auto grain boundary image; and (**d**) grain average misorientation image.

Figure 17 presents the optical microscopy and electron backscatter diffraction (EBSD) results for the alloy after 3.5% pre-stretching followed by aging at 155 °C. An analysis of the EBSD data reveals that the alloy exhibits an average grain aspect ratio of approximately 12.68 and a degree of recrystallization of 42.7%. The grain structure of aluminum alloys is primarily determined by the thermomechanical processing history prior to solution treatment. The grain characteristics are considered to be essentially consistent across all tested conditions. Therefore, the influence of the grain structure on the variation in mechanical properties among different samples can be considered negligible, allowing the discussion to focus primarily on the role of precipitation behavior. Pre-stretching deformation significantly alters the nucleation, growth, and competitive relationships of precipitation phases during subsequent aging by introducing high-density dislocations into the matrix. The TEM observations and statistical analysis along the <110>_Al_ zone axis (Figure 7 and Figure 8) show that increasing the pre-stretch deformation from 2.5% to 4.5% leads to a continuous refinement of the *T*_1_ phase. Specifically, its average diameter drops from 57.9 nm to 42.6 nm, accompanied by a sharp rise in the area density from 462 μm^−2^ to 908 μm^−2^. Observations along the <100>_Al_ zone axis reveal that the average diameter of the *θ*′ phase remained essentially constant at approximately 52 nm, while its number density increased linearly from 69 μm^−2^ to 123 μm^−2^. The *T*_1_ phase exhibits a significant lattice mismatch with the Al matrix, resulting in high interfacial energy and difficulty in uniform nucleation. Consequently, it strongly depends on crystal defects such as dislocations. The large number of dislocations introduced by pre-stretching provides dense, non-uniform nucleation sites for the *T*_1_ phase, significantly lowering the nucleation barrier. This promotes its precipitation with a higher density and smaller size. Notably, a competitive precipitation relationship exists between the *T*_1_ phase and the *θ*′ phase for Cu atoms. The stoichiometry of the *T*_1_ phase is Al_2_CuLi, and its precipitation process consumes Cu atoms. Under pre-stretching conditions, the extensive and rapid precipitation of the *T*_1_ phase inevitably consumes a large number of Cu atoms in the solid solution within the matrix, thereby inhibiting the growth behavior of the *θ*′ phase. This elucidates the observed increase in the number density of the *θ*′ phase following pre-stretching, despite the lack of commensurate growth in its size. The evolution of grain boundary precipitates arises as an indirect consequence of the aforementioned intragranular precipitation reactions. The enhanced precipitation of the *T*_1_ and *θ*′ phases within the grains consumes substantial quantities of Cu and Li solutes, leading to a localized solute depletion in the vicinity of the grain boundaries. This, in turn, suppresses the growth kinetics of grain boundary precipitates, resulting in finer precipitate sizes and a discontinuous morphological distribution.

The *T*_1_ phase is the most dominant strengthening phase in Al-Li alloy systems, with its strengthening contribution significantly exceeding that of the *θ*′ phase and the S′ phase. According to the precipitation strengthening theory, when the size of the precipitate phase is smaller than the critical shearing size (<55 nm) [31], the relationship between dislocations and precipitates follows a shearing mechanism, and the strength model is given as follows [32,33]:

(1) $$\sigma_{T_{1}}=\frac{1.211Md_{T_{1}}\gamma_{eff}^{3/2}}{t_{T_{1}}^{2}}\sqrt{\frac{bf_{T_{1}}}{\Gamma }}$$

When the size exceeds the critical shearing size, the strengthening mechanism transitions to Orowan bypassing, and the strengthening effect diminishes as the size increases. The strength model is given as follows [34,35,36]:

(2) $$\sigma_{T_{1}}=\frac{0.12MGb}{\sqrt{d_{T_{1}}t_{T_{1}}}}(\sqrt{f_{T_{1}}}+0.7\sqrt{\frac{d_{T_{1}}}{t_{T_{1}}}}f_{v}+0.12\frac{d_{T_{1}}}{t_{T_{1}}}f_{T_{1}}^{3/2})\ln(\frac{0.079d_{T_{1}}}{b})$$

where *γ_eff_* is the effective interfacial energy term (~0.107 J/m^2^), and *b* is the Burgers vector. Γ is the dislocation line tension (≈1/2*Gb*^2^), *M* is the average Taylor factor, and *t*_*T*1_, *d*_*T*1_, and *f*_*T*1_ are the average thickness, diameter, and volume fraction of *T*_1_ precipitates, respectively.

The Orowan strengthening contribution of the *θ*′ phase to the yield strength can be described as follows [33,37]:

(3) $$\begin{array}{l} \sigma_{\theta^{\prime }}=\frac{K_{\theta^{\prime }}}{0.931\sqrt{\frac{0.306\pi t_{\theta^{\prime }}}{f_{\theta^{\prime }}}}\sqrt{d_{\theta^{\prime }}}-\frac{\pi }{8}{\left(\sqrt{d_{\theta^{\prime }}}\right)}^{2}-1.061t_{\theta^{\prime }}}\\ K_{\theta^{\prime }}=(\frac{Gb}{2\pi \sqrt{1-v}})(\ln\frac{1.225t_{\theta^{\prime }}}{2b}) \end{array}$$

where *υ* is Poisson’s ratio (0.339), and *t*_*θ*′_, *d*_*θ*′_, and *f*_*θ*′_ are the average thickness, diameter, and volume fraction of *θ*′ precipitates, respectively.

While the S′ phase was occasionally observed, its volume fraction was minimal under the current pre-stretching conditions, and, thus, its influence on the mechanical properties is not discussed in detail. With increasing pre-stretching deformation, a higher dislocation density is introduced, thereby enhancing the contribution of strain hardening. Concurrently, this leads to a refinement in the size and an increase in the number density of the *T*_1_ phase. In the vicinity of the critical size, the strengthening contribution of the *T*_1_ phase is correlated with its diameter, thickness, and volume fraction, as described by the shearing model. Although the number density increases in the alloy with 4.5% pre-stretching, its diameter concurrently decreases. The findings reported by Xie et al. [38,39] also demonstrate that, under the T8 temper, the strengthening contribution of the *T*_1_ phase progressively diminishes with increasing pre-stretching. Consequently, the combined effects of precipitation strengthening and strain hardening result in comparable overall strength levels across the alloys investigated in this study.

Nevertheless, the intragranular distribution of the *T*_1_ and *θ*′ phases becomes more homogeneous and refined, effectively alleviating the stress concentration at the precipitate–matrix interface and delaying void nucleation and coalescence. Moreover, the grain boundary precipitates a transition from a continuous network to a discontinuous particulate morphology, substantially reducing the susceptibility to intergranular brittle fracture. These microstructural evolutions contribute to the enhanced ductility and toughness observed in alloys subjected to higher pre-stretching levels. The improvement in fatigue crack growth resistance can be attributed to the shrinkage of the cyclic plastic zone at the crack tip. The finely dispersed *T*_1_ phases effectively pin dislocations, suppressing the formation and propagation of cyclic slip bands and mitigating the cumulative damage per stress cycle [39]. Fractographic observations reveal that the fatigue striation spacing gradually decreases with increasing pre-stretching, providing indirect evidence for the reduced extent of crack tip blunting during cyclic loading.

### 4.2. Influence of Aging Temperature on Precipitation Competition and Boundary Characteristics

Aging temperature governs the nucleation, growth, and coarsening kinetics of precipitates. As the aging temperature increases, the average size of the *T*_1_ phase increases while its number density decreases. Aging at 165 °C promotes the coarsening of the *T*_1_ phase, leading to a reduced increment in strength. Furthermore, the grain boundary precipitates become noticeably coarser and exhibit a continuous distribution, providing a preferential path for crack propagation. This results in a decreased fracture toughness and an accelerated fatigue crack growth rate in the alloy. A fractographic analysis reveals that, after aging at 165 °C, the fracture surface tends to be flattened, with dimples becoming smaller and shallower, accompanied by an increased proportion of intergranular fracture. In contrast, aging within the temperature range of 145~155 °C achieves an optimal match between the size and number density of the *T*_1_ and *θ*′ phases. The intragranular precipitates exhibit a dispersed distribution, while grain boundary precipitates remain discontinuously distributed. This microstructure not only maintains a high strength but also effectively impedes crack propagation, thereby achieving an optimal balance between strength and toughness.

From the perspective of precipitation kinetics, the influence of aging temperature on precipitate evolution is primarily manifested in the following aspects: First, an increase in temperature enhances the diffusion capacity of atoms, thereby accelerating the nucleation and growth processes of precipitates. During the early stages of aging, a higher temperature facilitates rapid precipitate nucleation; however, as aging progresses, elevated temperatures promote the dissolution of smaller precipitates and the further growth of larger ones, leading to precipitate coarsening. Second, variations in the temperature affect the competitive relationships among different precipitate phases. Excessively high temperatures may induce the transformation of the *T*_1_ phase into other thermodynamically more stable phases, thereby compromising the strengthening effect.

Furthermore, the aging temperature exerts a significant influence on the structure of the precipitate–matrix interface. Aging at 155 °C maintains a coherent relationship between the precipitates and the matrix, characterized by low interfacial energy, which is conducive to enhancing the strength of the alloy. When the aging temperature is increased to 165 °C, the interface may lose coherency, transforming into a semi-coherent or incoherent interface. Although this may slightly improve the stability of the precipitates, it diminishes their capacity to impede the dislocation motion, thereby compromising the strengthening effect. The precipitation behavior at the grain boundaries is particularly sensitive to the aging temperature. Aging at 145 °C results in relatively fine, discontinuously distributed grain boundary precipitates. As the temperature increases, these grain boundary precipitates rapidly coarsen and eventually interconnect to form a continuous network. This continuous network structure provides a preferential path for crack propagation, significantly degrading the toughness and fatigue properties of the alloy [40]. Therefore, precise control of the aging temperature is crucial for balancing the intragranular strengthening and grain boundary precipitation behavior. To achieve an optimal combination of strength and toughness, the suitable aging temperature range for this alloy is identified as 145~155 °C.

## 5. Conclusions

In this study, the effects of pre-stretching deformation and aging temperature on the microstructural evolution and mechanical properties of an Al-Cu-Li alloy were investigated. The main conclusions are as follows:

(1) Increasing the pre-stretching deformation significantly enhances the number density and refines the size of the intragranular *T*_1_ and *θ*′ phases, while promoting a discontinuous distribution of grain boundary precipitates. This results in an improved plasticity and fracture toughness of the alloy without compromising its high strength, as well as a reduction in the fatigue crack growth rate.
(2) An increase in the aging temperature accelerates the coarsening of the *T*_1_ phase and reduces its number density, while inhibiting the precipitation of the *θ*′ phase. Grain boundary precipitates coarsen and exhibit a discontinuous distribution. Although the peak strength is not significantly affected, both the fracture toughness and fatigue crack growth resistance deteriorate markedly.
(3) The microstructure–property relationship of the alloy indicates that, by appropriately controlling the pre-stretching deformation (3.5~4.5%) and aging temperature (145~155 °C), the size, distribution, and grain boundary characteristics of the precipitates can be optimized. This enables a synergistic enhancement of high strength and excellent damage tolerance.

## Figures and Tables

**Table 1:** Chemical composition of the novel Al-Cu-Li alloy (wt.%).

Cu	Li	Mg	Ag	Zn	Mn	Zr	Al
4.04	1.29	0.44	0.42	0.39	0.30	0.10	Bal.

## Data Availability

The original contributions presented in this study are included in the article. Further inquiries can be directed to the corresponding authors.
